# Editorial: New insights into antitumor mechanisms based on breast cancer immune microenvironment

**DOI:** 10.3389/fgene.2023.1235699

**Published:** 2023-07-20

**Authors:** Yan Yao, Tianhua Wang, Changgang Sun

**Affiliations:** ^1^ College of First Clinical Medicine, Shandong University of Traditional Chinese Medicine, Jinan, China; ^2^ College of Traditional Chinese Medicine, Shandong University of Traditional Chinese Medicine, Jinan, China; ^3^ College of Traditional Chinese Medicine, Weifang Medical University, Weifang, China; ^4^ Department of Oncology, Weifang Traditional Chinese Hospital, Weifang, China

**Keywords:** Research Topic editorial, breast cancer, tumor immune microenvironment, molecular mechanism, immunotherapy

Breast cancer has the highest incidence of female cancers in the world. Immunotherapy has opened up a new era in the treatment of cancer, and the immune system’s role in breast cancer development and progression has been increasingly recognized. Nevertheless, the application of immunotherapy is also limited by the large heterogeneity between individual and molecular types. This interindividual variation focuses on the heterogeneity of the body’s immunity caused by the complex tumor immune microenvironment (TIM). The TIM has become a recent research hot spot, providing novel but valuable insight into tumor heterogeneity and clinical management, as well as multifaceted mechanisms of tumor progression. TIM is concerned to be the key factor to determine the benefit of immunotherapy, but the complex breast cancer immune microenvironment lacks in-depth and systematic characterization. Therefore, strengthening the understanding of the breast cancer immune microenvironment is not only the key to analyzing the immune regulation mechanism but also has guiding significance for improving the clinical efficacy of immunotherapy.

For this Research Topic, our focus is to delineate the TIM molecular map of breast cancer and conduct multi-omics studies on specific molecular regulatory mechanisms, so as to elucidate the characteristics of the breast cancer immune microenvironment, explore its specific properties, relationships, and dynamic changes, and help identify the biomarkers of immunotherapy.

High-throughput technologies are generating vast amounts of transcriptomic and genomic data, providing a snapshot for studying the tumor immune microenvironment. Bioinformatics analysis can assist researchers in more effectively analyzing and interpreting gene function and expression, identifying and predicting the impact of specific gene variations. This allows for a better understanding of the mechanisms underlying cancer development and aids in the design of effective treatment strategies. In our Research Topic, four papers were conducted based on cancer-related databases such as TCGA and GEO to build gene risk models for breast cancer, including cGAS-STING, Immunogenic cell death (ICD), circadian rhythm-related genes (CRRGs), and immune-related genes for triple-negative breast cancer. By analyzing with state-of-the-art algorithms, critical target genes for predicting the prognosis and treatment of breast cancer immune microenvironment were identified. This will contribute to the study of breast cancer immune infiltration and the development of more effective immunotherapy strategies.

Molecular epidemiology utilizes advanced techniques to measure the distribution of biological markers and, combined with epidemiological research methods, helps to elucidate the characteristics of the immune microenvironment of breast cancer at the molecular or genetic level and its related pathogenic processes. Wang et al. constructed a nomogram based on Chinese multicenter clinical data and the SEER database for rare breast cancer subtypes, such as metaplastic breast cancer (MBC), to predict postoperative overall survival (OS) in MBC patients, which contributes to personalized and precise discrimination of patients. Zhu et al. used a retrospective cross-sectional study to investigate the prevalence of hyperprolactinemia in Chinese premenopausal women with breast diseases and the relationship between hyperprolactinemia and different clinical characteristics, revealing the phenomenon that hyperprolactinemia is prevalent in Chinese premenopausal patients with breast diseases, especially in those with fibroepithelial tumors (FETs), suggesting that PRL levels may be moderately correlated with various breast diseases. Tan et al. conducted a prospective study using liquid biopsy and next-generation sequencing (NGS) to dynamically detect changes in circulating tumor DNA (ctDNA) and tumor burden and markers in the blood of advanced TNBC patients receiving ICIs, finding that CYP2D6 deficiency and increases in genes such as GNAS and BCL2L1 can predict the efficacy of ICIs in late-stage TNBC patients, and dynamic monitoring of ctDNA in late-stage TNBC patients may provide timely indicators of ICI treatment sensitivity. These studies contribute to the clinical guidance of immune therapy for different molecular subtypes of breast cancer.

Wilson et al. discussed the role of IL-1β in tumorigenesis by summarizing phase III data supporting the use of immunotherapy for TNBC and preclinical data supporting IL-1β inhibition as a potential therapeutic strategy. They further evaluated trials involving IL-1β in breast cancer and other solid tumor malignancies, providing a strong scientific rationale for the combination of IL-1β and immunotherapy in the neoadjuvant and metastatic setting for people with TNBC.

We hope that this Research Topic will advance our understanding of the complex interplay between immune cells and breast cancer cells in the breast cancer TIM, and facilitate the development of effective immunotherapeutic strategies for breast cancer. We would like to express our gratitude to all the authors who contributed to this Research Topic, as well as the reviewers who provided valuable feedback and helped ensure the quality of the articles.

In conclusion, we believe that the insights presented in this Research Topic will be beneficial in improving new breakthroughs in the field of breast cancer immunotherapy and ultimately improve patient outcomes.

